# Autophagy controls *Wolbachia* infection upon bacterial damage and in aging *Drosophila*

**DOI:** 10.3389/fcell.2022.976882

**Published:** 2022-10-10

**Authors:** Dávid Hargitai, Lili Kenéz, Muna Al-Lami, Győző Szenczi, Péter Lőrincz, Gábor Juhász

**Affiliations:** ^1^ Department of Anatomy, Cell and Developmental Biology, Eötvös Loránd University, Budapest, Hungary; ^2^ Institute of Genetics, Biological Research Centre, Szeged, Hungary

**Keywords:** autophagy, xenophagy, *Drosophila*, *Wolbachia*, wolbophagy

## Abstract

Autophagy is a conserved catabolic process in eukaryotic cells that degrades intracellular components in lysosomes, often in an organelle-specific selective manner (mitophagy, ERphagy, etc). Cells also use autophagy as a defense mechanism, eliminating intracellular pathogens *via* selective degradation known as xenophagy. *Wolbachia pipientis* is a Gram-negative intracellular bacterium, which is one of the most common parasites on Earth affecting approximately half of terrestrial arthropods. Interestingly, infection grants the host resistance against other pathogens and modulates lifespan, so this bacterium resembles an endosymbiont. Here we demonstrate that *Drosophila* somatic cells normally degrade a subset of these bacterial cells, and autophagy is required for selective elimination of Wolbachia upon antibiotic damage. In line with these, Wolbachia overpopulates in autophagy-compromised animals during aging while its presence fails to affect host lifespan unlike in case of control flies. The autophagic degradation of Wolbachia thus represents a novel antibacterial mechanism that controls the propagation of this unique bacterium, behaving both as parasite and endosymbiont at the same time.

## Introduction

Autophagy is a conserved process of eukaryotic cells in which cells isolate a portion of cytoplasm and degrade it by the lysosomal system (Jiang and Mizushima, 2014). During its most common form—macroautophagy—a double-membrane cistern grows around the cytoplasm destined for degradation and the newly formed autophagosome can subsequently fuse with lysosomes (Lőrincz and Juhász, 2020). Macroautophagy (hereafter simply autophagy) can be a selective process during which cell components and organelles—for example damaged mitochondria, ruptured lysosomes, or misfolded proteins—are targeted for degradation by selective autophagy receptors (Johansen and Lamark, 2011; Zaffagnini and Martens, 2016). Importantly, cells are not only able to protect themselves from their own damaged organelles but also from pathogens that have entered the cell. This type of selective autophagy is called xenophagy, referring to the foreign, invading target (Gomes and Dikic, 2014; Mao and Klionsky, 2017).

*Wolbachia pipientis* (Hertig and Wolbach, 1924) is a Gram negative rickettsial intracellular bacterium, which is one of the most common parasites on Earth (Anderson and Karr, 2001). Wolbachia infects approximately 40%–52% of terrestrial arthropods and it also parasitizes other invertebrates, such as human pathogenic filarial nematodes (Zug and Hammerstein, 2012; Weinert et al., 2015). Being an obligate intracellular parasite unable to live outside of a host, Wolbachia is classified as a reproductive parasite or endosymbiont (Charlat et al., 2003). To promote its own survival, the bacterium grants the host resistance against viruses and other pathogens (Teixeira et al., 2008; Martinez et al., 2014). For example, the spread of *Plasmodium falciparum*, the pathogen causing the dreaded disease malaria, is inhibited in Wolbachia infected mosquitos (Hughes et al., 2011). As infected mosquitos also gain resistance against viruses such as Zika or Dengue, Wolbachia carrying mosquitos have been released recently in areas hit by these epidemics (Frentiu et al., 2014; Callaway, 2016). These programs have turned out to be effective, so the potential role of Wolbachia in controlling the transmission of several mosquito-borne pathogens is in the focus of today’s research (Kamtchum-Tatuene et al., 2017). Although Wolbachia infection of such disease vectors benefits mankind, the situation is the opposite in case of pathogenic filarial worms. Elephantiasis and onchocerciasis causing filarial nematodes (which together affect more than 150 million people) are infected with Wolbachia, without which the host is unviable. Thus, anti-Wolbachia drugs have recently been developed to target the endosymbiont rather than the host to kill these pathogenic worms (Taylor et al., 2014; Johnston et al., 2021).

Wolbachia may enter the cell *via* receptor mediated endocytosis (White et al., 2017a) and restructure the endoplasmic reticulum to form Wolbachia containing vacuoles (WCVs) (Fattouh et al., 2019), but the subsequent fate of these is still unclear. As Wolbachia is normally absent from host cells, we hypothesized that its infection is controlled by autophagy. Recent studies suggested that Wolbachia can be either restricted or enhanced by autophagy in neotropical *Drosophila* species, fruit fly reproductive tissues or filarial worms (Voronin et al., 2012; Deehan et al., 2021; Strunov et al., 2022). We set out to clarify the connection between autophagy in somatic cells and Wolbachia, and to establish whether Wolbachia is indeed a substrate of selective autophagy.

In this work, we demonstrate that fruit fly somatic cells use selective autophagy to eliminate bacteria damaged by antibiotic treatment. In line with this, the aging-associated increase of Wolbachia numbers is more pronounced in autophagy mutant flies while the lifespan modulating effects of infection are lost.

## Materials and methods

### Fly work, antibiotics treatment

Initially, all of our fly strains were reared at RT on Tetracycline-HCL (0.25 mg/ml, Merck) containing standard medium for two subsequent generations to remove endogenous Wolbachia infection, if present (O'Shea and Singh, 2015). Wolbachia (Wmel CS2b) containing control (*w* ^*1118*^) strain (BL65286, RRID:BDSC_65286) was obtained from Bloomington *Drosophila* Stock Center, and we published the autophagy mutant *atg16[d67]* (Varga et al., 2016). Atg13 mutants (*atg13[d81]*) were previously described (Chang and Neufeld, 2009). By crossing Wmel CS2b infected females with males carrying balancer chromosomes or *atg16[d67]* or *atg13[d81]* mutant chromosomes, we successfully established Wmel CS2b infected autophagy mutant strains. For damaging Wolbachia, newly hatched L1 larvae were collected and reared on Tetracycline-HCL (0.25 mg/ml, Merck) containing food until late L3 stage. Starvation experiments were done by floating early L3 larvae on 20% sucrose for 3 h at room temperature.

### Immunostainings, western blots

Immunostainings of larval nephrocytes were performed as described (Lorincz et al., 2016) with at least three biological replicates. Briefly, samples were dissected in ice cold PBS, then fixed with 4% formaldehyde in PBS for 45′ at RT. Samples were 3x washed, incubated in 0.1% Triton X-100 in PBS (PBTX, 30′, RT), followed by blocking solution (5.0% FCS in PBTX). Samples were then incubated in the blocking solution containing primary antibodies (overnight, 4°C). Samples were rinsed 3×, washed in PBTX (3 × 10′ at RT), and incubated in blocking solution (30′ at RT). Samples were then incubated with secondary antibodies diluted in blocking solution for 3 h at RT. Washing steps were repeated, and DNA was stained using Hoechst 33,342 (Thermo). Samples were mounted in Vectashield (Vector). Western blots of larval and adult lysates were prepared as described (Pircs et al., 2012). Samples were homogenized, centrifuged, and supernatants containing equal amounts of proteins were separated by SDS-PAGE. Samples were then transferred to Immobilon-P PVDF membrane (Millipore). Membranes were blocked in 3% milk/TBS for 1 h at RT, washed 3 × 10′ in TBST (TBS+0.1% Tween-20). Blots were incubated with primary antibodies for 90′ at RT. Washing steps were repeated and blots were incubated with AP conjugated secondary antibodies (60’ at RT) followed by washing steps and colorimetric detection with nitroblue tetrazolium–5-bromo-4-chloro-3-indolyl phosphate (Sigma-Aldrich). Band intensities were evaluated using ImageJ (National Institutes of Health, Bethesda, MD).

### Chemicals and reagents

The following antibodies were used: rat anti-Atg8a 1:300; (Takats et al., 2013), mouse anti-Polyubiquitin FK2 1:200 (PW8810, ENZO Life Sciences, RRID:AB_10541840), rabbit anti-p62/Ref2p 1:2,000; (Pircs et al., 2012) (RRID:AB_2569199), mouse anti-tubulin 1:2,000 (AA4.3-s; DSHB, RRID:AB_579793), and rabbit anti-Wsp IF:1:200, WB:1200, (WSP-11S, Alpha Diagnostics Intl. Inc., United States). Secondary antibodies were Alexa Fluor 568 goat anti–rat, Alexa Fluor 568 goat anti–mouse, Alexa Fluor 488 goat anti–rabbit (all 1:1,000 Invitrogen) for immunofluorescence and AP-conjugated goat anti–rabbit, and anti–mouse 1:5,000, (EMD Millipore) for Western Blots.

### Microscopy and statistics

**FIGURE 1:**
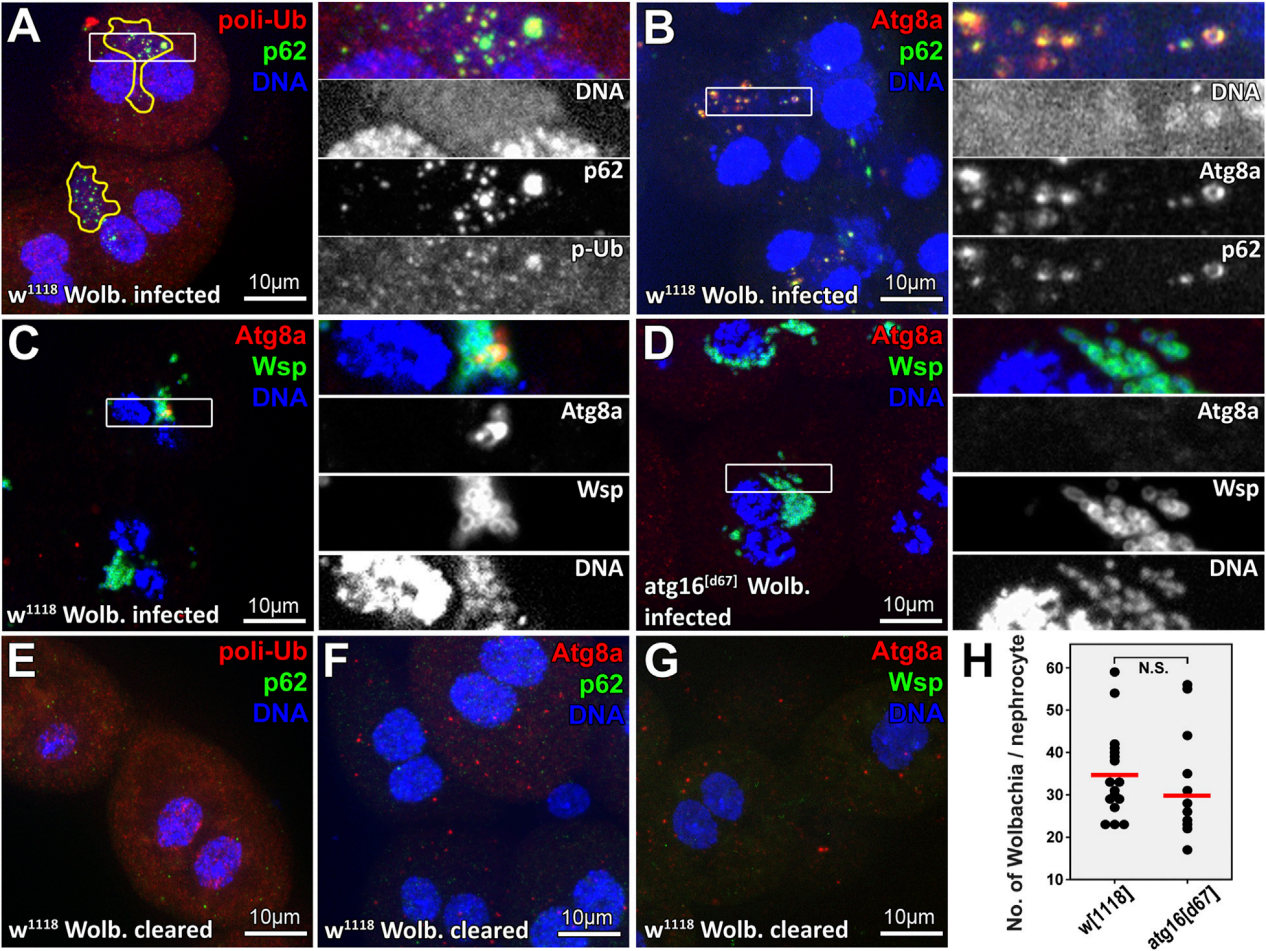
A subset of Wolbachia cells colocalize with selective autophagy markers in larval nephrocytes. DNA staining reveals clusters of bacteria (enclosed by yellow lines in panel **(A)** near the two brighter nuclei of larval *Drosophila* garland nephrocytes. Within these clusters, large polyubiquitin-p62 or Atg8a-p62 double positive structures can be observed (panels **A, B** respectively), indicating that some of these bacteria are targeted by selective autophagy. Wolbachia surface protein (Wsp) staining confirms the identity of Wolbachia captured within Atg8a positive autophagosomes **(C)**. No Atg8a positive membranes can be seen in *atg16* mutant nephrocytes **(D)**. Elimination of Wolbachia by rearing animals on tetracycline containing food prevents the appearance of large polyubiquitin-p62 or Atg8a-p62 double positive structures in nephrocytes **(E–G)**. **(H)** Quantification of data in C, **(D)** *n* = 30 control (*w* ^*1118*^) and *n* = 24 *atg16[d67]* cells (from eight to eight animals), red line indicates median of data.

**FIGURE 2:**
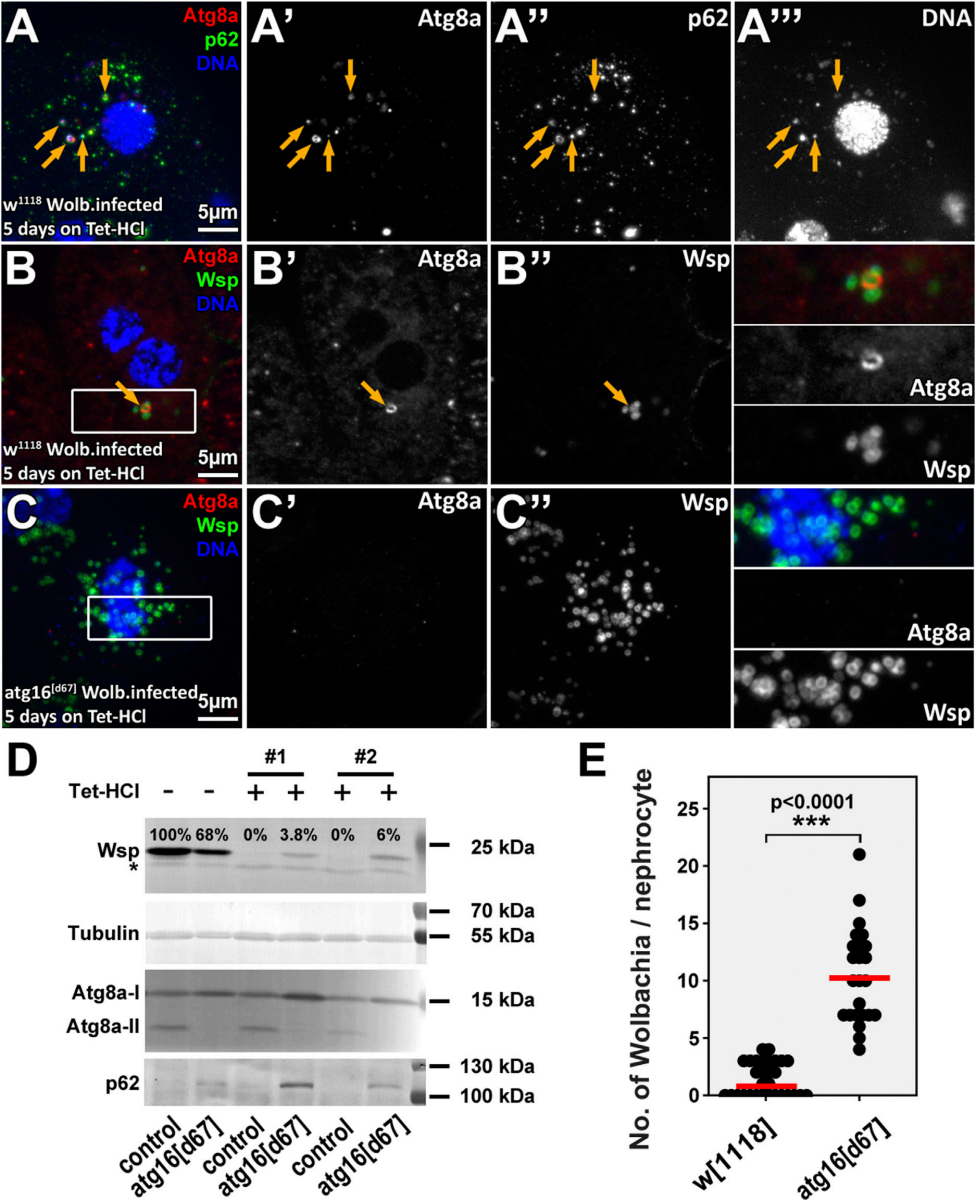
Wolbachia cells are eliminated by autophagy in tetracycline treated animals. Rearing animals on tetracycline containing food for a short period results in dispersal of normally perinuclear Wolbachia clusters both in control and *atg16* mutant nephrocytes **(A–C)**. However, control nephrocytes contain (probably dying) Wolbachia cells that are positive for autophagic markers **(A,B)**, unlike *atg16* mutant nephrocytes, in which more bacteria persist and no Atg8a positive membranes can be seen **(C,E)**. Yellow arrows point to Atg8a-p62-DNA triple positive **(A)** or Atg8a-Wsp double positive **(B)** structures. Western blot experiments **(D)** using larval lysates reveal that Wsp can be still detected in lysates of *atg16* mutant larvae by the time by which the control animals become Wolbachia free. The absence of lipidated, autophagosome associated Atg8a (Atg8a-II) and the accumulation of p62 in *atg16[d67*] lysates confirm that autophagy is indeed disrupted in mutants and no autophagic membranes are generated. Importantly, the levels of both forms of Atg8a and p62 remained the same in Wolbachia infected and antibiotic treated control lysates, indicating that Wolbachia has no substantial effect on general autophagy in larvae. Asterisk shows an unspecific band, #1, #2 indicate two independent experiments. **(E)** Quantification of data in B, **(C)** *n* = 17 control (*w* ^*1118*^) and *n* = 11 *atg16[d67]* cells (from four to four animals), red line indicates median of data.

Dissected whole mount 30-day-old adults were fixed in 3.2% PFA, 1% glutaraldehyde, 1% sucrose, and 0.028% CaCl_2_ in 0.1 N sodium cacodylate pH 7.4, overnight at 4°C. Samples were then postfixed in 0.5% osmium tetroxide for 1 h and in half-saturated aqueous uranyl acetate for 30 min at RT, dehydrated in a graded series of ethanol, and embedded in Durcupan (Fluka) according to the manufacturer’s recommendations. Semi-thin sections were cut to identify organs and tissues. Then 70-nm sections were stained in Reynolds lead citrate and viewed on a JEM-1011 transmission electron microscope (Jeol) equipped with a Morada digital camera (Olympus) using iTEM software (Olympus). Fluorescent images were captured using an AxioImager M2 microscope (Zeiss) equipped with an ApoTome2 grid confocal unit and a Plan-Apochromat 63×/1.40 Oil objective, an Orca Flash 4.0 LT + sCMOS camera (Hamamatsu Photonics). Wolbachia numbers in controls and mutants were counted manually from unmodified single confocal images from the nuclear plane and data were evaluated by SPSS17 (IBM). T-tests were used for comparing datasets that showed normal distribution (Figures 1B,C, Supplementary Figure S3), and U tests for comparing samples that contained at least one variable showing non-Gaussian data distribution (Figures 2B,C, Supplementary Figure S4).

### Survival tests

We compared the life span of infected and non-infected *atg16* mutant and control female flies in similar settings in three independent experiments as before (Varga et al., 2016). Males were not tested to avoid potential bias as in some cases Wolbachia reduces the viability of males (Sheeley and McAllister, 2009). We used the same control and mutant stocks with or without Wolbachia. Vials containing control and mutant flies were kept next to each other in the same tray on the same shelf of a 25°C thermostat. Survival of *atg13[d81]* animals was not tested as this mutation causes late larval/pupal lethality*.* (Chang and Neufeld, 2009). Survival of flies were compared using Breslow-Wilcoxon survival test.

## Results

### Wolbachia containing vacuoles are positive for selective autophagy markers

We routinely use the large binucleate *Drosophila* larval garland nephrocyte to study the endolysosomal system and larval fat body to study autophagy (Lorincz et al., 2016; Lorincz et al., 2017a; Lorincz et al., 2017b; Lorincz et al., 2019). During our experiments, we often observed small DNA positive dots near the polyploid nuclei of nephrocytes in some of our control strains, which were observed less frequently in fat cells. Since 30% of fruit fly stocks maintained in the Bloomington *Drosophila* Stock Center are infected with Wolbachia (Clark et al., 2005), we assumed that the often cloud-like, weak DNA staining labels this bacterium in our stocks. Our assumptions were supported by the fact that tetracycline treatment effectively removed these DNA positive structures from nephrocytes. This gave us motivation to study the possible role of autophagy in controlling Wolbachia in larval nephrocytes, in which autophagic activity is normally very low (Lorincz et al., 2019).

Therefore, we obtained a control fruit fly stock (*w* ^*1118*^) infected with Wolbachia (strain Wmel CS2b) from Bloomington *Drosophila* Stock Center. Wolbachia has been shown to increase ubiquitin positive puncta numbers in infected insect cells, which was suggested to represent modulation of proteolysis in host cells (White et al., 2017b). We first stained larval garland nephrocytes for ubiquitin and the selective autophagy receptor p62 (Nezis et al., 2010; Johansen and Lamark, 2011). We indeed found more ubiquitin puncta in infected cells, but the dots were detected within the large perinuclear bacterial DNA positive clusters in these cells, and the ubiquitin signal overlapped with p62 (Figures 1A,E). Moreover, we could detect the colocalization of bacterial DNA, p62 and Atg8a (Figures 1B,F), indicating that it is not Wolbachia that increases ubiquitination of host structures, but rather the host targets bacterial cells for degradation. To prove this, we obtained a commercial antiserum against Wolbachia surface protein (Wsp) to confirm that the appearing Atg8a positive autophagic structures contain Wolbachia and that this cannot happen in *atg16* mutant cells (Figures 1C,D,G,H).

### Autophagy promotes the clearance of damaged Wolbachia

The findings that only a small subset of Wolbachia were targeted for autophagy in controls and that bacteria numbers did not differ significantly between control and autophagy mutant nephrocytes indicated that autophagy is normally unable to eliminate Wolbachia infection even though it eliminates a subset of bacteria. Importantly, mitochondria and rickettsial bacteria (including Wolbachia) share a common ancestor (Thrash et al., 2011; Ferla et al., 2013) and autophagy is used to eliminate damaged or unwanted mitochondria *via* selective mitophagy (Palikaras et al., 2018). By following this logic, we hypothesize that host cells likely eliminate a Wolbachia cell if it is damaged and thus possibly harmful. As neither a selective marker of Wolbachia damage nor a selective Wolbachia damage inducer is available, we turned to antibiotics. Therefore, we inhibited protein synthesis in Wolbachia by feeding larvae with Tetracycline-HCl (Tet-HCl) containing food and tested if the clearance of (possibly dying) Wolbachia is accompanied by its autophagic degradation. Antibiotic treatment led to the scattering of the originally dense Wolbachia cluster and p62-positive bacterial cells were indeed captured inside autophagosomes (Figures 2A,B, Supplementary Figures S1A,B). Even growing, unsealed autophagic membranes could be detected around the bacteria (Supplementary Figure S2). Importantly, proper clearance of Wolbachia was clearly inhibited in *atg16* or *atg13* mutants (Figures 2D,E, Supplementary Figures S1C,D,S2,S3), which was confirmed by Western blotexperiments (Figure 2D, Supplementary Figures S4A,B). These data indicate that autophagy contributes to the clearance of damaged/dying Wolbachia and that Tet-HCl does not alter autophagy by itself. Importantly Atg8a positive bacteria could be observed in *atg13* mutant cells, in line with Atg8a lipidation proceeding in these cells (Nagy et al., 2015). To check whether elevated autophagy can decrease Wolbachia load we compared the amount of Wsp in lysates from well fed and starved larvae. Since starvation induced autophagy had no effect on Wolbachia load in any of these genotypes (Supplementary Figure S4C), we conclude that only damaged/injured bacteria are subject to autophagic degradation.

### Wolbachia propagation is out of control in the absence of autophagy during aging

**FIGURE 3:**
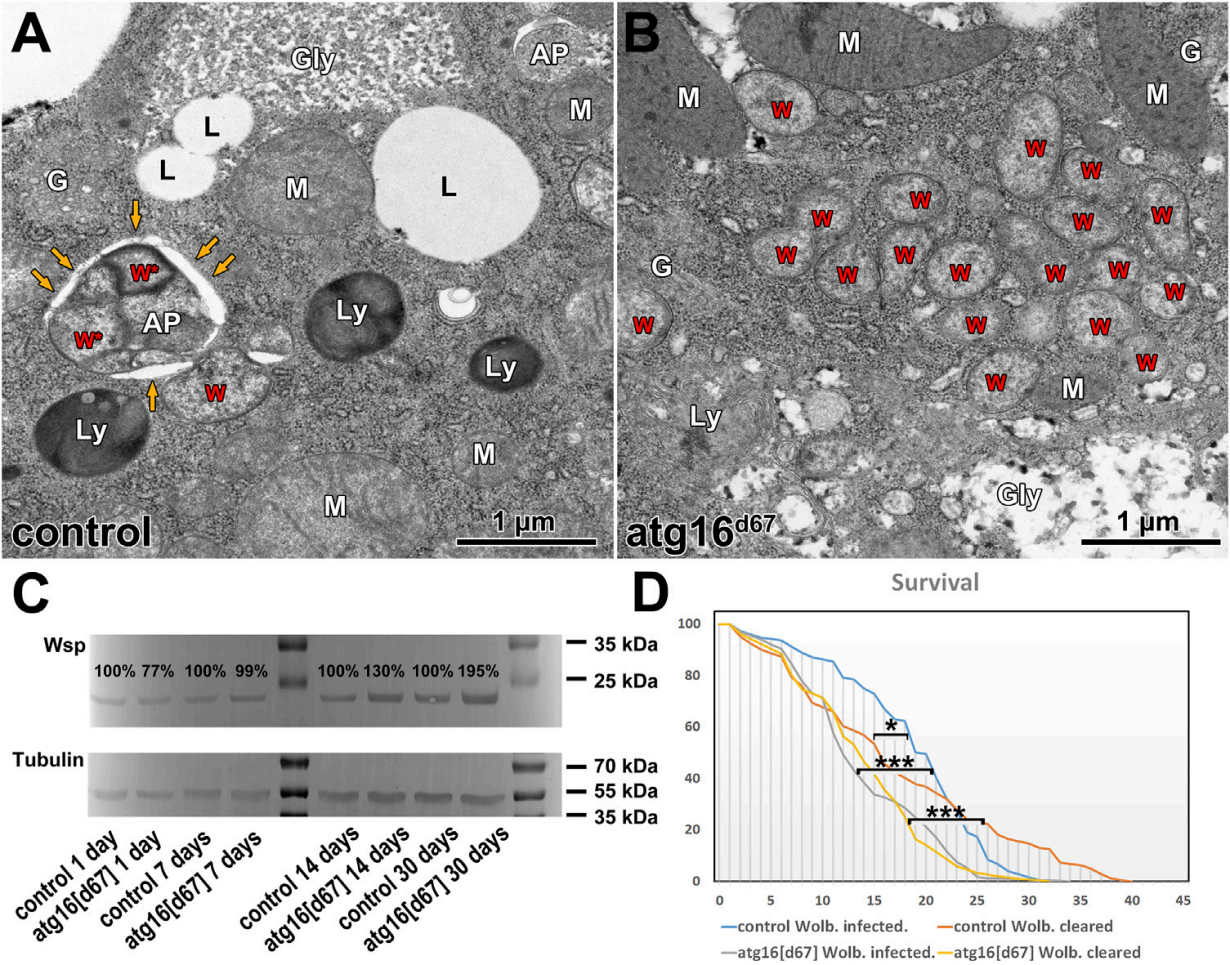
Wolbachia in aging animals. Ultrastructure of fat cells of 30 days old adults reveal Wolbachia inside autophagosomes **(A)**, and large Wolbachia clusters in atg16 mutants **(B)** in the absence of autophagosomes. W: Wolbachia, AP: autophagosome, yellow arrows point to the outer limiting membrane of an AP with Wolbachia inside (W*). Ly: lysosome, G: Golgi apparatus, Gly: glycogen granule, L: lipid droplet, M: mitochondrion. Western blot from adult lysates **(C)** reveal that the amount of detectable Wsp gradually increases in atg16 mutants compared to controls. Lifespan curves of Wolbachia infected *versus* cleared controls and atg16 mutants **(D)** show that atg16 mutants live shorter than controls (***: *p* <0.001) and their lifespan is unaffected by infection status (p: NS). Wolbachia infection initially increases the survival of control flies, (*: *p* = 0.042) but this effect declines over time.

It has been shown in many organisms including flies that autophagy declines during aging, and inhibition of autophagy shortens lifespan (Juhász et al., 2007; Simonsen et al., 2008; Maruzs et al., 2019; Aman et al., 2021). Aging correlates with the accumulation of damaged organelles and misfolded proteins in the cells. Based on these, we hypothesized that damaged Wolbachia cells would be continuously generated during aging, which would be eliminated by autophagy. In line with this, we observed Wolbachia inside autophagosomes of fat cells in aged 30 days old flies, in contrast with large Wolbachia groups and no autophagosomes in similarly aged autophagy mutant flies (Figures 3A,B). We next turned to western blot experiments and lifespan analyses to determine the relationships between autophagy, aging and Wolbachia. Strikingly, Wolbachia load gradually increased, doubling in *atg16* mutants by age 30 days compared to controls (Figure 3C).

### Wolbachia fails to extend lifespan in autophagy mutant animals

Wolbachia can alter the host’s lifespan, fitness and stress tolerance, but these effects are sometimes inconsistent: both lifespan extension and shortening have been reported in different conditions (Maistrenko et al., 2016). Therefore, we tested the longevity of control and *atg16* mutant females with and without Wolbachia. We observed that Wolbachia infected controls survived better at the beginning of the experiment, however, survival declined after 3–4 weeks, and all infected animals eventually died before remaining Wolbachia free controls did. *Atg16* mutants lived shorter than controls did and Wolbachia infection status had no effect on survival (Figure 3D). These results suggest that modulation of the host’s fitness by Wolbachia requires autophagy in adult flies.

## Discussion

*Wolbachia pipientis* is one of the most (if not the most) abundant intracellular bacterium on Earth. While it infects almost half of terrestrial arthropods and several worm taxa and its biomedical importance is clear, its interactions with the host cell have remained largely unexplored, which is especially true for autophagy. This gave us motivation to test whether infected *Drosophila* cells can get rid of Wolbachia *via* autophagy, utilizing autophagy null mutant flies for the first time. Indeed, a subset of bacteria were positive for markers of selective autophagy, indicating that this process likely plays a role in controlling Wolbachia.

Wolbachia is closely related to the endosymbiotic alphaproteobacterium, from which the mitochondrion evolved (Thrash et al., 2011; Ferla et al., 2013). Wolbachia also grants several benefits for the host, among which resistance against pathogens may be most important (Teixeira et al., 2008; Martinez et al., 2014). Indeed, Wolbachia carrying mosquitos have been released recently in areas hit by Zika or Dengue to prevent the spread of viruses (Frentiu et al., 2014; Callaway, 2016).

However, unlike the mitochondrion, Wolbachia remains quite independent of the host and Wolbachia genes were not transferred into the nucleus. This is probably because its acquisition occurred much later, by the time eukaryotes had developed powerful mechanisms for prevention of lateral gene transfer (Markov and Zakharov, 2006). This means that Wolbachia must export its proteins into the host’s cytoplasm to alter the metabolism and reproduction of the host, so its actions resemble both a parasite and an endosymbiont (Charlat et al., 2003). As only a small subset of Wolbachia cells is targeted by autophagy, and we saw no obvious inhibition of general autophagy in Wolbachia infected animals, these suggest that Wolbachia does not suppress autophagy in the host, in contrast to several well-known pathogens that evolved many different effective strategies to avoid autophagy (Huang and Brumell, 2014).

We hypothesize that Wolbachia containing vacuoles either are recognized as cell-organelles or are not detected at all by the defense systems of the host cell. This means that cells only recognize Wolbachia if it becomes “visible,” which happens when Wolbachia loses its “camouflage,” perhaps by getting damaged that may somehow pose a threat to the cell’s normal functioning. Inhibition of bacterial protein synthesis using tetracycline indeed triggered Wolbachia clearance at least in part *via* autophagy, and this clearance was partially inhibited in the autophagy mutant (probably dead bacteria disintegrate over time anyway, even in the absence of autophagy). Unfortunately, it is not possible to detect damaged Wolbachia. Since inducing autophagy by starvation has no effect on Wolbachia load, it suggests that healthy bacteria can evade degradation by host cells even if bulk autophagy is induced.

The hallmark of selective xenophagy is that bacteria are first tagged with ubiquitin and subsequently recognized by ubiquitin-binding protein adaptors, which then results in the capture of these bacteria into autophagosomes (Gomes and Dikic, 2014; Mao and Klionsky, 2017; Hu et al., 2020). This process appears to be similar to the best characterized mitophagy pathway, during which the E3 ligase parkin ubiquitinates dozens of mitochondrial outer membrane proteins to trigger selective mitophagy (Goodall et al., 2022). Our studies show that Wolbachia can be indeed targeted for autophagic degradation by ubiquitin and p62, although the molecular background and ubiquitination targets of Wolbachia and/or Wolbachia-containing vacuolar membranes remain to be elucidated. A common theme of bacterial autophagy may be the rupture of bacterium-containing vacuoles, which results in the exposure of carbohydrates attached to the inner membrane surface that recruit galectins to promote ubiquitylation of possibly multiple targets, including lipopolysaccharide (Goodall et al., 2022). Ubiquitin ligases are indeed important for targeting *Salmonella*, *Streptococcus* and other pathogens for autophagic degradation (Sharma et al., 2018), but in these cases the bacterial target proteins are unknown. Moreover, in the case of the gram positive bacterium *Mycobacterium tuberculosis,* the Rv1468c surface protein directly binds ubiquitin to promote host xenophagy (Chai et al., 2019).

The autophagy machinery can contribute to bacterial clearance in two ways: cells can use selective macroautophagy to eliminate them by xenophagy (Gomes and Dikic, 2014; Mao and Klionsky, 2017; Hu et al., 2020) or *via* non-canonical autophagic pathways involving conjugation of Atg8 to single membranes (CASM) (Durgan et al., 2021; Deretic and Lazarou, 2022). These later processes are independent of the Atg13/Atg1/ULK1 complex but depend on the Atg8 conjugation system including Atg16 (Durgan et al., 2021). During these, Atg8 proteins are conjugated to phosphatidylethanolamine (PE) or phosphatidylserine (PS) and subsequently targeted to single membranes, such as bacteria containing phagosomes (Durgan et al., 2021). Atg8a targeting to WCVs in *atg13* mutants raises the possibility that CASM may also contribute to elimination of Wolbachia. As we see double-membrane autophagosomes containing Wolbachia during aging and growing, unsealed autophagic isolation membranes are engulfing bacteria, probably macroautophagy is more critical for eliminating damaged bacteria. It is also possible that inhibiting macroautophagy induces compensatory CASM in atg13 mutants. Future studies will be necessary to elucidate the exact contributions of all these molecular mechanisms for WCV clearance.

Unexpectedly, Wolbachia load was slightly lower in autophagy mutant larvae than in controls based on our WB experiments. Nutrient availability and host diet heavily affect Wolbachia propagation (Serbus et al., 2015). We believe that this may explain the initially lower Wolbachia titer in autophagy mutants, which are very sensitive for starvation: their anabolic pathways rely more on food because they are unable to properly recycle nutrients from the cytosol (Juhász et al., 2007).

Since autophagy decline and increasing number of damaged organelles are hallmarks of aging (Aman et al., 2021), we tested whether autophagy is required to keep Wolbachia under control during aging. Autophagy can indeed eliminate Wolbachia in controls, while it overpopulates the autophagy mutant host. Autophagy defective flies are often viable but short-lived due to decreased stress tolerance and accelerated aging (Juhász et al., 2007; Varga et al., 2016; Jipa et al., 2021). Importantly, Wolbachia infection has no effect on the lifespan of *atg16* mutants. These results suggest that autophagy is somehow necessary for the benefits of Wolbachia infection for the host. We favor a model in which autophagy functions as a quality control mechanism by removing damaged Wolbachia and preventing its over proliferation during aging, similar to mitophagy.

Our study shows that Wolbachia can be captured but it is normally not eliminated by autophagy. In contrast, autophagy is necessary for its timely elimination upon bacterial injury (in this case, inhibition of bacterial protein synthesis). Wolbachia is undoubtedly an endosymbiont in filarial nematodes (including pathogenic ones, which together affect more than 150 million people) as without the bacterium the worm dies. Recently, anti-Wolbachia drugs have been developed to fight these pathogenic worms (Taylor et al., 2014; Johnston et al., 2021), and autophagy-inducing drugs such as rapamycin treatment decrease Wolbachia number in pathogenic worms (Voronin et al., 2012).

**FIGURE 4:**
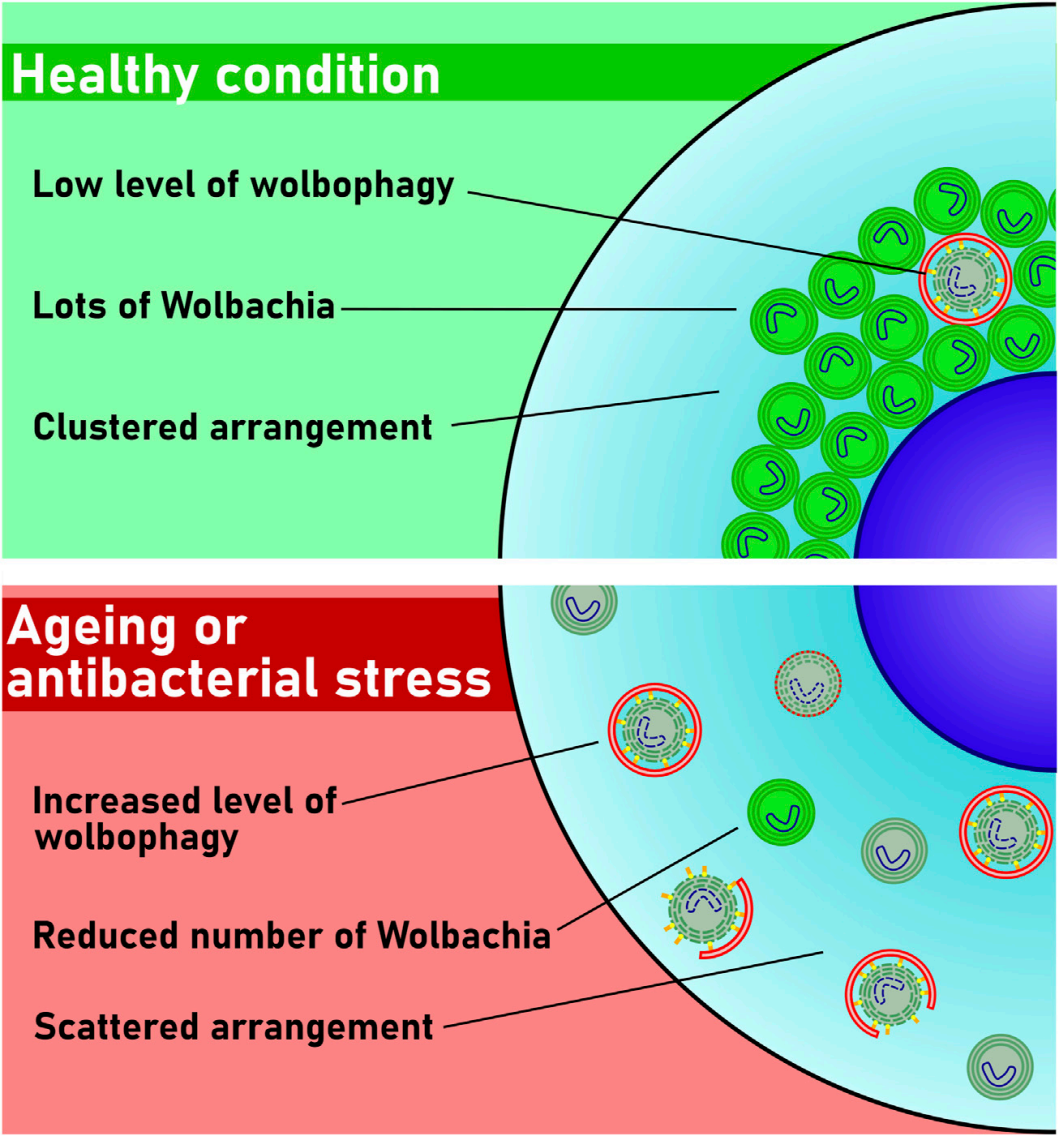
Model of wolbophagy. Only a few bacteria are damaged and thus eliminated in healthy cells. The number of damaged Wolbachia increases during aging or upon antibiotic stress. These damaged bacteria are then targeted by autophagy, and perhaps also by conjugation of Atg8 to single membranes (CASM) for elimination by wolbophagy.

Taken together, we show that the autophagic elimination of Wolbachia represents a novel antibacterial mechanism to control the propagation of this bacterium. Importantly, as Wolbachia seems to behave both as a parasite (resembling invading bacteria that are targeted by xenophagy) and endosymbiont (resembling mitochondria that are targeted by mitophagy) at the same time, we propose that its autophagic elimination deserves a new term: wolbophagy (Figure 4).

## Data Availability

The original contributions presented in the study are included in the article/Supplementary Material, further inquiries can be directed to the corresponding authors.
