# Supplementary material for: Autophagy controls Wolbachia infection upon bacterial damage and in aging Drosophila
Source: Front Cell Dev Biol. 2022 Oct 10;10:976882. doi: 10.3389/fcell.2022.976882 (PMC9589277; doi:10.3389/fcell.2022.976882)
Supplement: Supplementary file 1 [file Table1.DOCX]

Supplementary Material

**Supplementary Figure 1.** **Wolbachia clusters disperse upon tetracycline treatment.** Clusters of Wolbachia can be seen near the polyploid nuclei of nephrocytes of control (A) and *atg16* (B) mutant animals that are raised on control food. Feeding animals with Tet-HCl containing food results in the dispersal of these clusters and Wolbachia cells become scattered in the cytosol.

**Supplementary Figure 2.** **Wolbachia associated Atg8a positive membranes (possibly isolation membranes) can be detected in tetracycline treated animals.** Yellow arrow points to an Atg8a positive membrane associated to a Wsp positive bacterium.

**Supplementary Figure 3.** **Wolbachia cells are eliminated by autophagy in tetracycline treated animals.** Rearing animals on tetracycline containing food for a short period results in dispersal of normally perinuclear Wolbachia clusters both in control (A, D) and *atg13* (B, E) mutant nephrocytes. Interestingly, nephrocytes in both genotypes contain (probably dying) Wolbachia cells that are positive for the autophagic marker Atg8a (B, E, see also the insets) indicating that Atg8a lipidation proceeds in these cells. This raises the possibility that a non-canonical autophagic pathway involving conjugation of Atg8 to single membranes (CASM) contributes to wolbophagy. C: Quantification of data in A, B. n = 12 control (*w^1118^*) and n=12 *atg13[d81]* cells (from 4-4 animals), red line indicates median of data. F: Quantification of data in D, E. n = 8 control (*w^1118^*) and n=8 *atg13[d81]* cells (from 3-3 animals), red line indicates median of data.

**Supplementary Figure 4.** **Additional Western blot data.** An experimental replicate of Figure 2D is shown in panel A. Panel B shows the quantification of WB data in panel A and Figure 2D. n = 4 control (*w^1118^*) and n=4 *atg16[d67]* independent lysates from multiple animals in all cases that were raised on control food. n = 5 control (*w^1118^*) and n=5 *atg16[d67]* independent lysates of animals raised on Tet-HCl containing food. (C) Western blot experiments using larval lysates reveal that the level of the Wolbachia-specific protein Wsp remains the same upon starvation both in control and *atg* mutant animals.
