# Supplementary figures and images for: Autophagy controls Wolbachia infection upon bacterial damage and in aging Drosophila
Source: Front Cell Dev Biol. 2022 Oct 10;10:976882. doi: 10.3389/fcell.2022.976882 (PMC9589277; doi:10.3389/fcell.2022.976882)

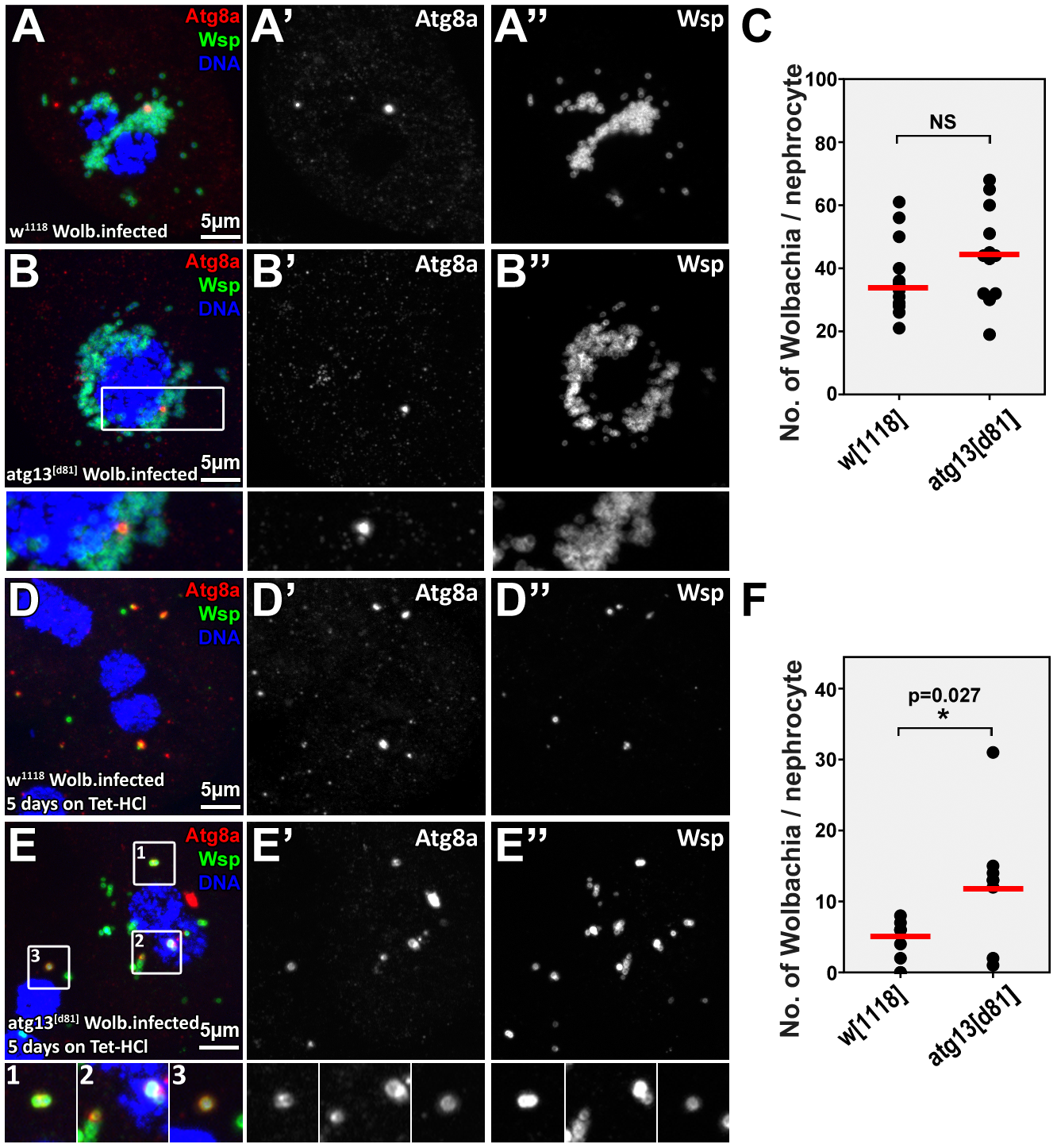

Supplement: Supplementary file 2 [file Image3.TIF]

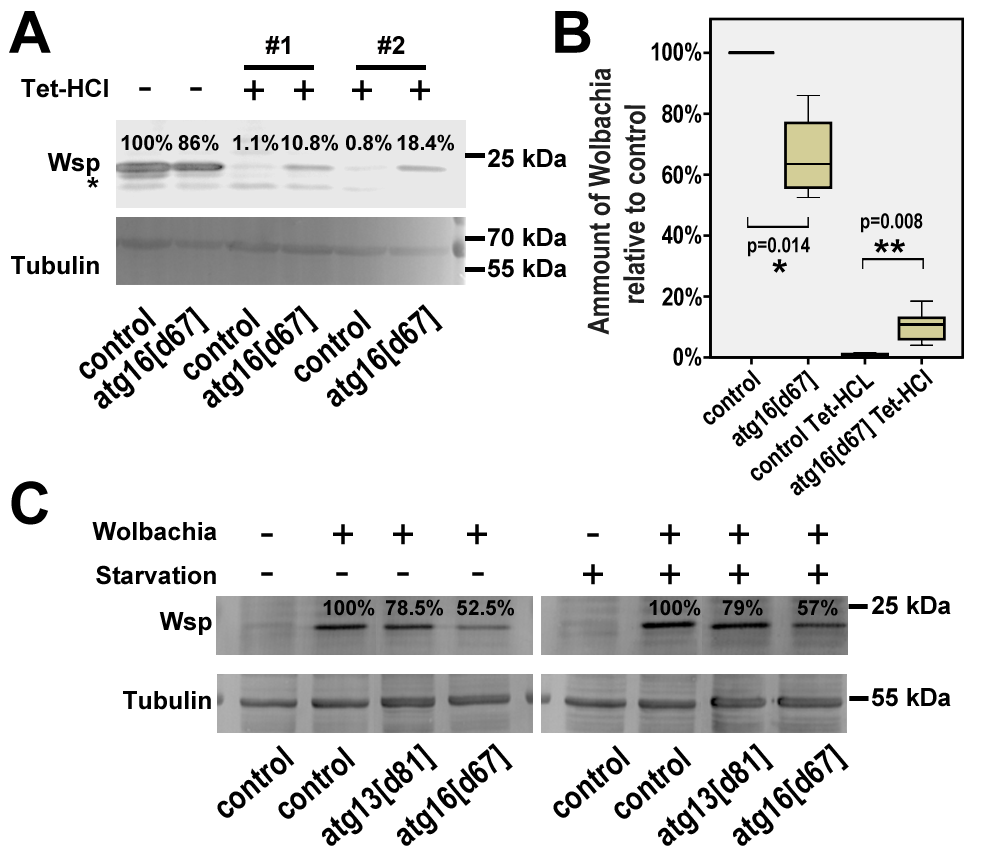

Supplement: Supplementary file 3 [file Image4.TIF]

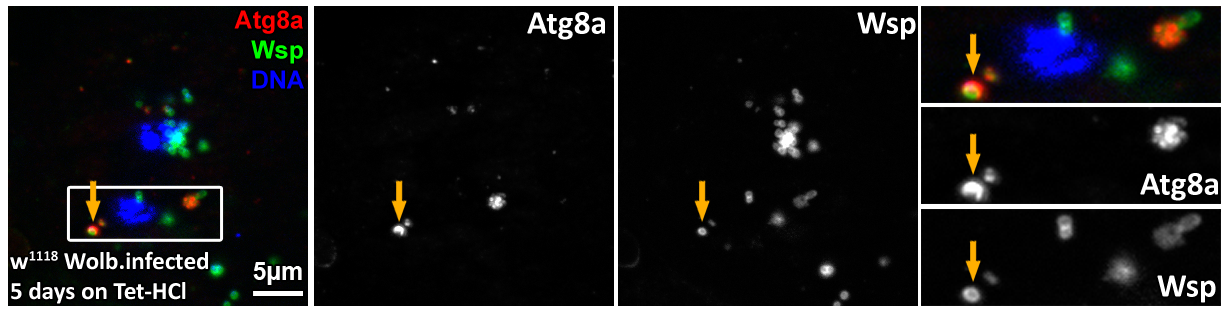

Supplement: Supplementary file 4 [file Image2.TIF]

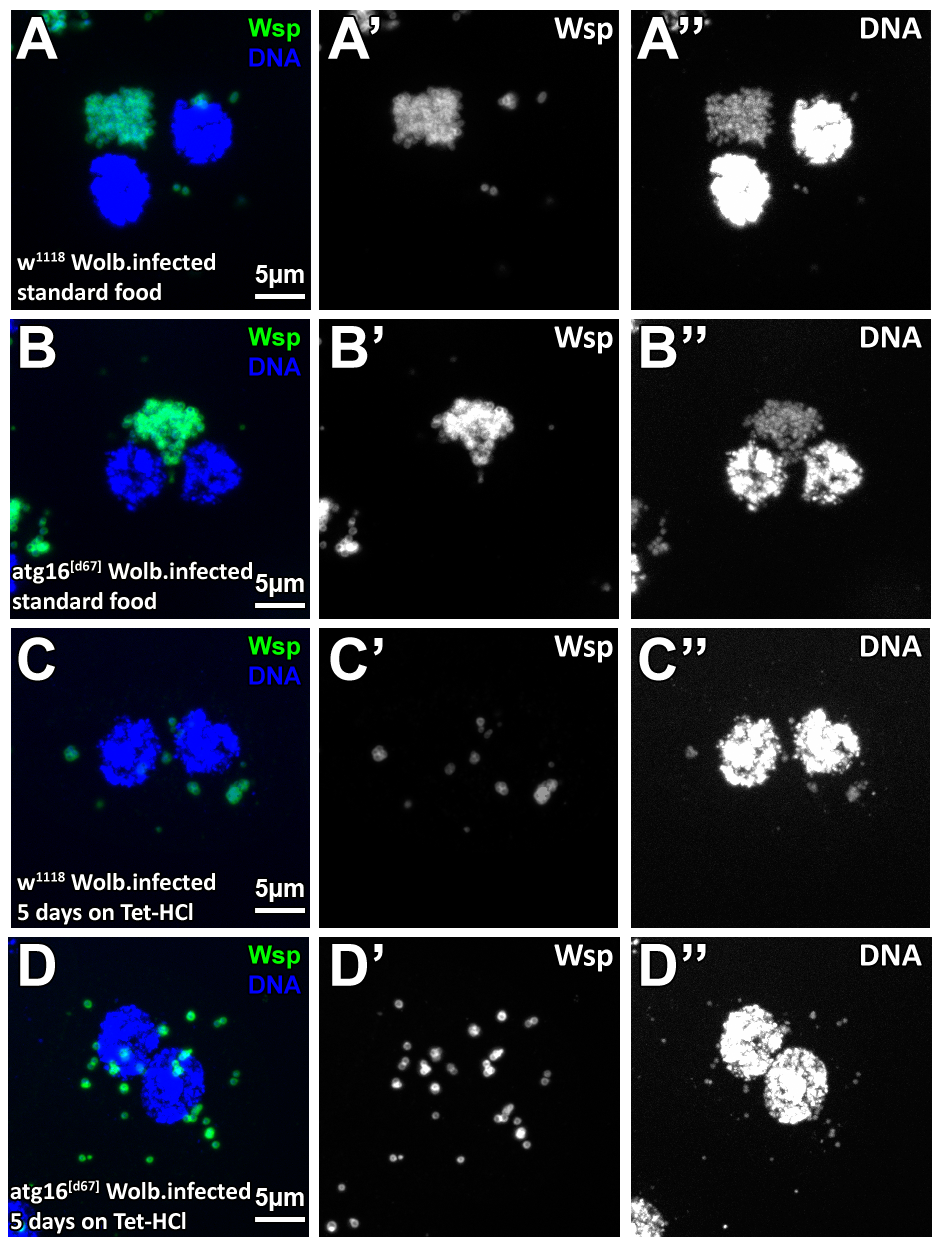

Supplement: Supplementary file 5 [file Image1.TIF]
